# A Hybrid Structure to Improve Electrochemical Performance of SiO Anode Materials in Lithium-Ion Battery

**DOI:** 10.3390/nano14141223

**Published:** 2024-07-19

**Authors:** Jian Yu, Chaoran Zhang, Xiaolu Huang, Leifeng Cao, Aiwu Wang, Wanjun Dai, Dikai Li, Yanmeng Dai, Cangtao Zhou, Yaozhong Zhang, Yafei Zhang

**Affiliations:** 1 Shenzhen Key Laboratory of Ultraintense Laser and Advanced Material Technology, Center for Intense Laser Application Technology, College of Engineering Physics, Shenzhen Technology University, Shenzhen 518118, China; caoleifeng@sztu.edu.cn (L.C.); wangaiwu@sztu.edu.cn (A.W.); daiwanjun@sztu.edu.cn (W.D.); lidikai@sztu.edu.cn (D.L.); daiyanmeng@sztu.edu.cn (Y.D.); zhoucangtao@sztu.edu.cn (C.Z.); 2 State Key Laboratory of Metal Matrix Composites, Shanghai Jiao Tong University, Dong Chuan Road No. 800, Shanghai 200240, China; chrzhang@sjtu.edu.cn; 3 Key Laboratory for Thin Film and Microfabrication Technology of the Ministry of Education, School of Electronics, Information and Electrical Engineering, Shanghai Jiao Tong University, Dong Chuan Road No. 800, Shanghai 200240, China; huangxiaolu@sjtu.edu.cn; 4 School of Materials Science and Engineering, Shanghai Jiao Tong University, Dong Chuan Road No. 800, Shanghai 200240, China

**Keywords:** lithium-ion batteries, silicon monoxide, electrospinning, carbon nanofibers

## Abstract

The wide utilization of lithium-ion batteries (LIBs) prompts extensive research on the anode materials with large capacity and excellent stability. Despite the attractive electrochemical properties of pure Si anodes outperforming other Si-based materials, its unsafety caused by huge volumetric expansion is commonly admitted. Silicon monoxide (SiO) anode is advantageous in mild volume fluctuation, and would be a proper alternative if the low initial columbic efficiency and conductivity can be ameliorated. Herein, a hybrid structure composed of active material SiO particles and carbon nanofibers (SiO/CNFs) is proposed as a solution. CNFs, through electrospun processes, serve as a conductive skeleton for SiO nanoparticles and enable SiO nanoparticles to be uniformly embedded in. As a result, the SiO/CNF electrochemical performance reaches a peak at 20% the mass ratio of SiO, where the retention rate reaches 73.9% after 400 cycles at a current density of 100 mA g^−1^, and the discharge capacity after stabilization and 100 cycles are 1.47 and 1.84 times higher than that of pure SiO, respectively. A fast lithium-ion transport rate during cycling is also demonstrated as the corresponding diffusion coefficient of the SiO/CNF reaches ~8 × 10^−15^ cm^2^ s^−1^. This SiO/CNF hybrid structure provides a flexible and cost-effective solution for LIBs and sheds light on alternative anode choices for industrial battery assembly.

## 1. Introduction

Energy density and cycle life are two vital factors to judge the performance of lithium-ion batteries (LIBs). Motivated by the large application potential of LIBs in portable electronic devices, electric vehicles, hybrid electric vehicles, and grid-scale storage, the improvement of these factors is being extensively investigated [1,2,3,4,5,6]. Accordingly, much effort has been devoted to the development of high-capacity electrode materials [7]. It was found that the commercial graphite may not be a proper anode material due to the low capacity (372 mAh g^−1^). Instead, silicon-based materials attracted much attention because of the high theoretical specific capacity (~4200 mAh g^−1^ for Si, ~2400 mAh g^−1^ for SiO and ~1680 mAh g^−1^ for SiO_2_), natural abundance, environmental benignity, and relatively low working potential [8,9,10]. Table 1 lists various Si-based anode materials (Si, SiO and SiO_2_) with the related factors in lithium-ion batteries. As for properties alone, silicon stands out among the electrode materials with ultrahigh theoretical specific capacity (4200 mAh g^−1^) [11,12,13]. However, particle pulverization, active materials detaching, and a weak solid electrolyte interface induced by a huge volumetric expansion during cycling would gravely endanger the performance and safety [14,15]. The option of SiO_2_ has mild volume fluctuation (~100% the volume expansion) during lithiation/delithiation processes that increases safety; however, the deficient diffusion coefficient of Li-ion leads to obstacles being widely used [16]. For SiO, it is comparatively favored because, firstly, its relatively high theoretical capacity, stable cyclic performance and low volumetric variation have been verified under various conditions [17,18,19,20]. Secondly, the lithiation of SiO can activate the formation of Li_2_O and Li_4_SiO_4_ as an inert layer, where the volumetric change can be hindered and the cyclability can be ameliorated [21,22,23,24]. Further, SiO has a microstructure with Si nanoparticles distributed in a matrix of Si suboxides; therefore, as an active material, SiO has a large Li insertion capacity, which gives it advantages in stability and unique electrical properties [25,26]. In view of this, SiO as a promising anode material draws increasing attention [13,26,27,28,29,30,31,32,33].

Despite the fortes of SiO, there are still some drawbacks, such as volume change, poor electrical conductivity and cycling instability, hindering it from commercial applications [42,43,44]. To address these issues, various strategies, such as doping and composites, have been developed, as shown in Table 2. For instance, Jae Hyun Kim et al. fabricated a boron-doped SiO using solution dopant during thermal disproportionation, which exhibited 947 mAh g^−1^ at 0.5 C rate and excellent capacity retention of 93.3% over 100 cycles [45]. Cui’s group presented a new nanocomposite as the lithium source, where homogeneously dispersed active Li_x_Si nanodomains were embedded in a robust Li_2_O matrix, delivering remarkable air compatibility and cycling performance [44,46]. Wang et al. synthesized a SiO/Fe_2_O_3_ composite by mechanical milling and the material shows superior electrochemical performance [47]. These efforts made some progress on the problem of volume change and improved electrochemical performance effectively, but the problem of low electrical conductivity and volume change still remain.

Recently, carbon-and-SiO-combined composites consisting of various carbonaceous precursors and special structural designs were developed to overcome these issues and volume expansion [27,48,49,50]. With good conductivity, stable chemical properties, small volume dilation and low costs, carbon-based materials were frequently combined with SiOx to avoid direct contact with electrolytes so that the side effects would not impact the total performance of electrodes [51,52]. These composites had carbon coating and harnessed porosity functioning as a protector of SiO particles and to ensure the conductivity of intact composites, simultaneously, and therefore were able to prevent cycling stability loss [53,54]. Zheng’s group reported the positive effect of single-walled carbon nanotubes as a conductive additive on the performance amelioration of SiO/C anodes. The 3D conductive skeleton of single-walled carbon nanotubes was found to be well covered with all the SiO/C primary particles [16]. Li et al. investigated the interfacial instability between SiO@C and LiPF_6_/dimethyl carbonate electrolyte, which would help design high-performance silicon monoxide-based anode materials and corresponding compatible electrolytes in LIBs [55]. Dou et al. developed a three-dimensional binder network, polydopamine-grafted cross-linked polyacrylamide, to build a durable anode for LIBs; it maintains 591 mAh g^−1^ after 300 cycles with a 94% capacity retention of initial capacity after activation even at a high current density of 1 A g^−1^ [56]. Although this is effective, the miscellaneous procedures and expensive raw materials during SiO-based anode synthesis are huge barriers for scalability, with much less commercialization. A flexible, highly efficient, scalable and low-cost process for SiO-based anode materials is highly desired.

Electrospinning is a facile and novel technology that can generate a cobweb-like analogue structure on a microcosmic scale. It is favored for LIB fabrication due to the easy operation requirements, convenient utility set-up and ecologically friendly features [57,58]. The mechanical milling method is also commonly used in industry and can offer SiO materials with excellent cycle performance by using the manipulation of nanosized SiO and content of SiO_2_ [59]. Herein, in this study, commercial SiO as the raw material was ball-milled and used to fabricate SiO/PAN nanofibers via electrospinning. After being carbonized in the high-temperature inert atmosphere, the SiO/CNF composite could be obtained directly. A number of advantages were demonstrated on the as-prepared nanofibrous composite. (1) The nanofibrous carbon skeleton could load the SiO nanoparticles evenly, which excluded particulate reunion and elevated the usage rate of active materials. (2) It strengthened the overall conductance performance of SiO/CNF, which improved the electrochemical activity of SiO. (3) CNF-covered SiO particles could effectively restrict the volume expansion of SiO during cycling, which was in favor of the stability of SiO electrochemical properties. The resulting SiO/CNF exhibited high Coulombic efficiencies, stable cycling performance and excellent rate capability as an anode material for LIBs, demonstrating its significant potential for commercial applications.

## 2. Materials and Methods

### 2.1. Materials Preparation

Micro-sized SiO (99.99%) particles utilized in this research were provided by Shanghai Jiuding Chemical Co., Ltd. (Shanghai, China) The SiO powder was ball-milled under Ar atmosphere at a speed of 800 rpm for 12 h. The weight ratio of powders to balls was 1:10. N,N′-dimethylformamide (DMF) was purchased from Shanghai Aladdin Biochemical Technology Co., Ltd. (Shanghai, China). Polyacrylonitrile (PAN, Mw = 90,000) was procured from Macklin Inc. (Shanghai, China).

### 2.2. Synthesis of SiO/CNF

The ball-milled SiO powder was initially dissolved and dispersed in a DMF solution through 6 h of stirring followed by 6 h of ultrasonication to achieve uniform dispersion. PAN powders were then added to the DMF solution containing SiO nanoparticles, and the mixture was stirred at 60 °C for an additional 12 h to obtain a viscous precursor solution suitable for electrospinning.

A syringe containing 10 mL of the SiO/PAN/DMF solution was connected to a spinneret jet with a needle with an inner diameter of 0.6 mm. The electrospinning procedure was conducted under 20–30% relative humidity, with the needle tip kept 15 cm away from the rectangular aluminum foil collector and the voltage stabilized at 15 kV. The precursor solution was dispensed at a rate of 0.6 mL h^−1^. Various electrospun nanofibers were produced by adjusting the ratios between SiO and PAN, resulting in samples labeled SiO/PAN-1, SiO/PAN-2, and SiO/PAN-3 with ratios of 1:4, 1:2, and 1:1, respectively.

Following electrospinning, the as-fabricated SiO/PAN fibers were preoxidized at 230 °C in air for 2 h, ramping at 2 °C min^−1^, to stabilize their structure. Subsequently, carbonization was performed at 800 °C for 2 h in a N2 atmosphere, ramping at 5 °C min^−1^. The resulting carbonized samples were denoted as SiO/CNF-1, SiO/CNF-2, and SiO/CNF-3 for SiO/PAN-1, SiO/PAN-2, and SiO/PAN-3, respectively.

### 2.3. Structural Characterization

The microscopic characterizations of a series of SiO/PAN nanofibers were captured by the field emission scanning electron microscope (FE-SEM, Carl Zeiss Ultra 55, Oberkochen, Germany). Transmission electron microscope (TEM, Talos F200X, Waltham, MA, USA) was used to collect the exquisite structures and elemental dispersion of SiO/CNF. The crystalline features of the collected samples were detected via a powder X-ray diffractometer (XRD, D8 Advance with Cu Kα radiation of 0.15418 nm). Raman spectrometer (Via Reflex Raman using 532 nm diode laser with 2400 L/mm grating) was utilized for analysis of the sample’s ingredients. Fourier-transformed infrared spectroscopy (FTIR, VERTEX 70, Billerica, MA, USA) spectra were collected for indications of structural evolution concerning the SiO/PAN and SiO/CNF. Thermogravimetric spectroscopy (TGA, Pyris 1, Waltham, MA, USA) was carried out with a ramping rate of 5 °C min^−1^ in flowing air. X-ray photoelectron spectroscopy (XPS, AXIS UltraDLD, Kyoto, Japan, Al target) was used to analyze the formation, and the corresponding spectra were fitted using the XPSPEAK41 program and Shirley-type background.

### 2.4. Electrochemical Measurements

The active materials were mixed with acetylene black (conductive agent) and sodium carboxymethyl cellulose (CMC, binder) with an 8:1:1 mass ratio. The homogeneously blended slurry was gained after sufficient agitation. Hereafter, copper foil (current collector) was brushed with the liquid slurry and the coated copper foil was dried at 110 °C for 12 h in a vacuum oven. The loading mass of the electrode was 1.8 g. The CR2016 coin cells were assembled in a high-purity Ar gas-filled glovebox. Lithium foil was used as the counter electrode. A total of 1 M LiPF_6_ was dissolved in ethyl carbonate/dimethyl carbonate (EC/DMC, 1:1, in volume) as the electrolyte, in which 10 wt% of fluoroethylene carbonate (FEC) was an additive. Polypropylene (Celgard) separator was fabricated to space out the electrode and lithium foil. Galvanostatic charge/discharge was measured in the 0.01–3 V (vs. Li^+^/Li) by a Land tester (Land CT 2001A, Wuhan, China). Cyclic voltammogram (CV) tests and electrochemical impedance spectrometry (EIS) tests were executed using an electrochemical workstation (CHI 760E). CV profiles were collected under a sweep rate of 0.05 mV s^−1^ in the voltage range of 0.01–1.2 V (vs. Li^+^/Li) and EIS data were conducted in the frequency range of 10^−2^ to 10^5^ Hz. All the electrochemical profiles were collected at room temperature.

## 3. Results and Discussion

**Figure 1:**
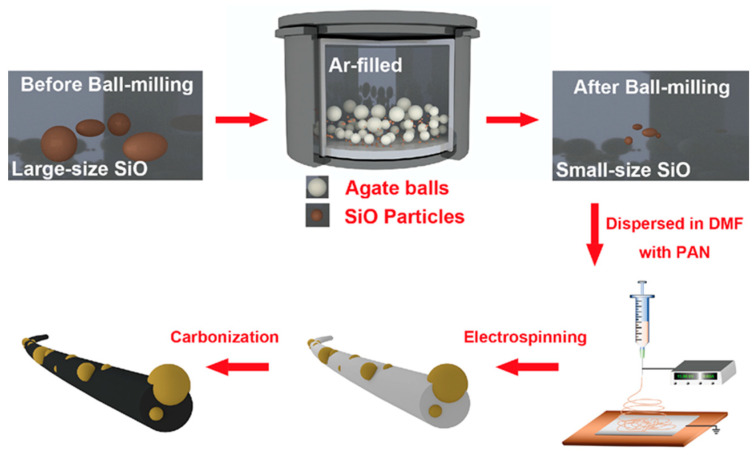
Scheme of SiO/CNF preparation.

A schematic diagram of the SiO/CNF synthetic process is shown in Figure 1. The particulate diameter of commercial SiO powders was minimized by inert atmosphere ball-milling, and the ball-milled SiO powders were dispersed in the DMF solution while adding the PAN to prepare the precursor solution for electrospinning. Whereafter, the SiO/PAN nanofibers were fabricated using solutions with different components; hence, the SiO/PAN nanofibers were in situ carbonized into SiO/CNF by high-temperature heat treatment, where SiO particles were embedded into a carbonaceous nanofibrous skeleton. At this point, the SiO/CNF used for LIBs was acquired.

Figure S1 exhibits the morphology of SiO particles before and after ball-milling. A clear observation can be noticed from the SEM images that the ball-milling process can reduce particle size discernibly. For the commercial SiO powders, as shown in Figure S1a–c, the large-sized particles agglomerate together easily, whose diameter ranges from 0.67 to 11.16 μm and average diameter equals 3.04 μm. After sufficient ball-milling process, the particles size was reduced adequately as exhibited in Figure S1d–f. Obviously, the SiO particles after ball-milling appear to be more delicate and homogeneous compared to those before ball-milling. Furthermore, SiO particles almost possess a spherical shape. The diameter distribution histogram shown in Figure S1h illustrates that the particle diameter ranges from 82 to 387 nm, and the average diameter is 206 nm. These results prove that the ball-milling process warrants particle-size diminution and superior particulate homogeneity, assuring the viability of SiO particles deployed in the LIBs.

**Figure 2:**
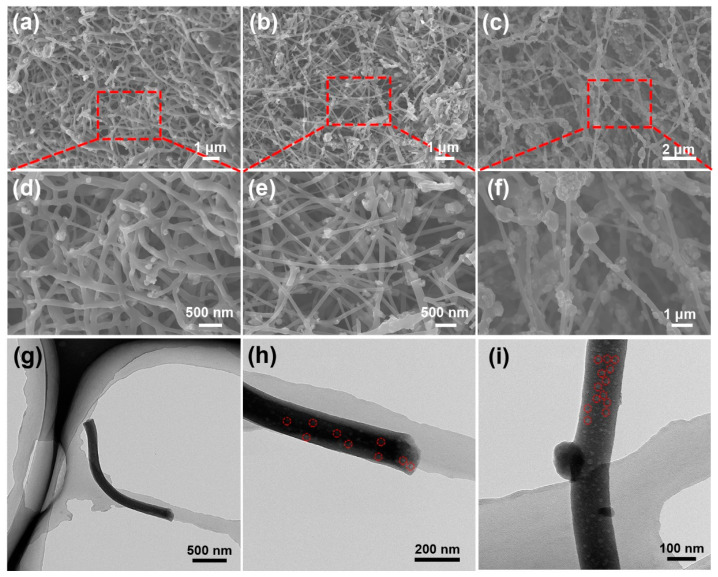
SEM images of SiO/CNF with different SiO contents: (**a**,**d**) SiO/CNF-1, (**b**,**e**) SiO/CNF-2 and (**c**,**f**), and SiO/CNF-3. (**g**–**i**) TEM images of SiO/CNF-1.

The SEM was used to investigate the morphology of SiO/CNF with different SiO contents. As shown in Figure 2, SiO particles are embedded in CNF for each SiO/CNF sample, yet there are still distinguished aspects between various SiO/CNF samples with different SiO components. In Figure 2a,d, SiO particles in SiO/CNF-1 with the lowest SiO content are dispersed with the best homogeneity and particles can be evenly distributed in the CNF skeleton. The CNF skeleton functions as the main framework while the SiO particles are embedded sparsely and uniformly but integrated judiciously with CNF. With the increasing amount of SiO powders, SiO particles look denser and some of them commence to reunite with each other in some areas, as demonstrated in Figure 2b,e. Most of the SiO particles can still be uniformly dispersed in the CNF skeleton, and only a few large particles extrude from CNF network. As shown in Figure 2c,f, the SiO/CNF-3, which possesses the highest concentration of SiO among these three samples, signified an uneven dispersion of SiO particles in a wide range. The CNF skeleton was almost fully embedded with SiO particles, and severe agglomeration of SiO particles appeared in many regions. Compared with the SiO/CNF-2, the number of SiO particles detached from the CNF framework increases markedly and most aggregated SiO particles reside in the voids generated by nanofibers. Preliminarily, with the increase in SiO concentration, the SiO in CNF tends to evolve from a homogeneous distribution to agglomeration gradually, and from a uniform distribution in CNF to separation from the CNF skeleton. Given this widely implemented knowledge, that embedding SiO in the CNF is beneficial for the electrochemical properties, choosing the right proportion of ingredients is of vital significance. Consequently, the sample with the best constituent also performs well across all samples with electrochemical properties.

Based on this, TEM was used to further observe SiO/CNF-1 with the best distribution of SiO on the CNF skeleton. Figure 2g–i exhibit the TEM images of SiO/CNF-1. It can be seen from the TEM images that SiO particles are evenly distributed in the interior and surface of the fiber. As shown in the marked areas in Figure 2h,i, most of the particles are wrapped by CNF, and a few particles are embedded on the surface of CNF. The overall CNF skeleton can provide an effective conductive network for SiO particles. During the charging and discharging process, the volume variation in a great majority of SiO particles can be effectively restricted with the mounting inside CNF which possesses considerable mechanical strength. Meanwhile, the conductivity of a few SiO particles embedded onto the surface of CNF can be enhanced with the help of numerous CNF interwoven networks.

**Figure 3:**
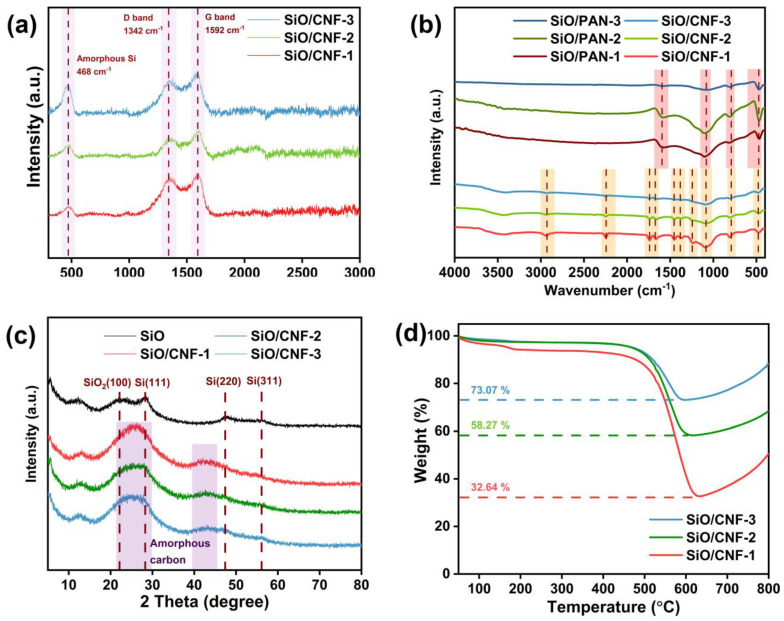
(**a**) Raman spectroscopy of various SiO/CNF samples; (**b**) FTIR spectra of samples with different constituents before and after carbonization; (**c**) XRD spectra of SiO and various SiO/CNF and (**d**) TGA curves of SiO/CNF with different contents.

In order to characterize the structure of carbonized SiO/CNF, three SiO/CNF samples were analyzed by Raman spectroscopy. Figure 3a shows the Raman spectroscopy of various SiO/CNF samples. It can be seen from the Raman spectroscopy that the characteristic peak positions of SiO/CNF-1, SiO/CNF-2 and SiO/CNF-3 are highly similar. The peak located at 468 cm^−1^ is in accord with the characteristic peak of amorphous Si; this is consistent with the literature results [60]. Furthermore, the pronounced peaks that appear at 1342 and 1592 cm^−1^ correspond to the disordered carbon structure (the D band) and the graphitic structure (the G band), respectively. D band and G band correspond to the defects in the structure of carbonaceous materials and the in-plane vibration of sp^2^ hybrid carbon atoms in carbon material, respectively [61]. For different samples, with the increase in the SiO content, the peak intensity ration of Si peak to C peak increases gradually. Therefore, the change in SiO content in the SiO/CNF composite material can be reflected by the change in the peak intensity ration of Si peak to C peak, demonstrating that the Raman spectroscopy results are in agreement with our design.

In order to determine the structural changes in SiO/PAN and SiO/CNF, the FTIR measurements were conducted on the samples before and after carbonization. As is shown in Figure 3b, before carbonization, a broad peak can be seen at 3400 cm^−1^. It should be the –OH functional group caused by water in the environment. The characteristic peaks located at 2923, 1445 and 1266 cm^−1^ correspond to the symmetric stretching of the –CH_2_ in the PAN skeleton [62]. The peaks located at 2244 cm^−1^ can be ascribed to the stretching vibration of C≡N of PAN. The peak appearing at 1740 cm^−1^ should be related to the C=O bond. The peak at 1664 cm^−1^ is supposed to correspond to the stretching vibration caused by C=C/N. Additionally, the peaks located at 1368 and 469 cm^−1^ can be assigned to the stretching vibration of –CH on PAN and oscillation modes of SiO_4_, respectively. The peaks at 1090 and 800 cm^−1^ belong to the Si-O-Si asymmetric and symmetric stretching vibrations, respectively [63]. For the carbonized samples (SiO/CNF-1, SiO/CNF-2 and SiO/CNF-3), the characteristic peaks located at 1590 and 1247 cm^−1^ correspond to the C=C bond and symmetric stretching of CH_2_ bond. Similar to the FTIR spectra of samples before carbonization, a pair of absorption peaks located at 1077 and 794 cm^−1^ correspond to the asymmetric vibration and symmetric vibration of Si-O-Si, respectively. Additionally, the peak at 474 cm^−1^ corresponds to the vibration structure of SiO_4_. Therefore, it can be concluded that the functional groups and structure of nanofibers before and after carbonization have obvious changes. The structures of nanofibers evolve from the occupation of C≡N and –CH_2_ groups before carbonization to the possession of only –CH_2_ and C=C groups afterward, whilst the Si-O-Si and SiO_4_ inside the SiO structure remain unchanged. These results indicate that the cyclization, dehydrogenation, oxidation and carbonization processes have already occurred during the transformation of PAN nanofibers to CNF nanofibers [58].

Figure 3c shows the XRD spectra of pure SiO and a series of SiO/CNF samples. It can be seen from the spectra that for the pure SiO, the diffraction peaks located at 28.2, 47.4 and 56.0° correspond to (111), (220) and (311), respectively [64]. Considering that the commercial preparation method of SiO is to deposit the mixture of Si and SiO_2_ by heating at a high temperature, the diffraction peak (100) of SiO_2_ can be seen at 22.1°. Although the characteristic peaks of Si and SiO_2_ can be seen in the XRD spectra, the peaks are not sharp, and most of them are wide peaks. As for the SiO/CNF samples, the two gentle broad peaks at 25 and 42° in the XRD pattern correspond to amorphous carbon. In addition, the appearance of amorphous carbon and SiO peaks indicates that the nanofiber composites achieve a good combination of SiO and CNF.

To further determine the mass percentage of SiO in each SiO/CNF sample, the TGA measurement was utilized to analyze the content of SiO in different samples from 50 to 800 °C at a 5 °C min^−1^ heating rate under air atmosphere. As shown in Figure 3d, the curves show a weight loss of 67.36 wt%, 41.73 wt% and 26.93 wt% for SiO/CNF-1, SiO/CNF-2 and SiO/CNF-3, respectively, which is caused by carbon oxidation. Thus, the SiO contents of SiO/CNF-1, SiO/CNF-2 and SiO/CNF-3 are 32.64 wt%, 58.27 wt% and 73.07 wt%, respectively. Furthermore, it can be indicated that the sample’s mass tended to increase after 630 °C, which was related to the combination of SiO with oxygen in the air to generate SiO_2_ at a high temperature.

**Figure 4:**
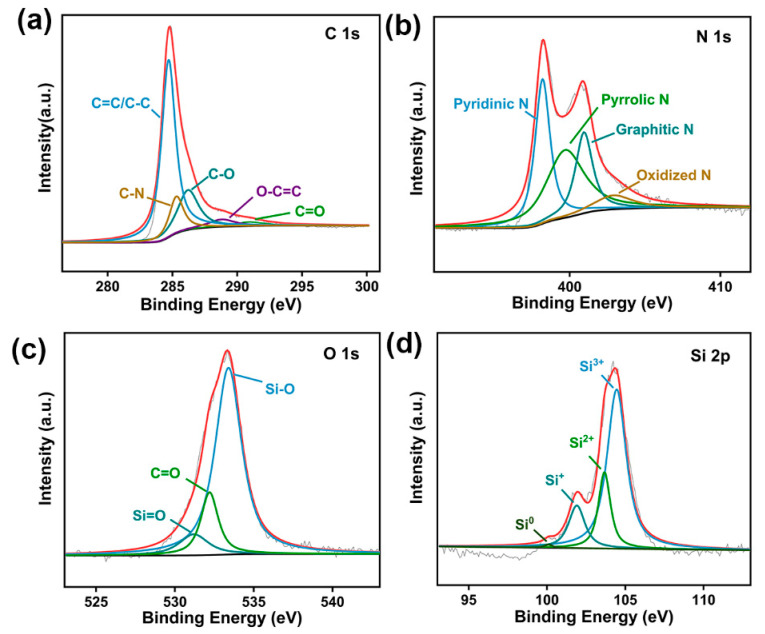
XPS of SiO/CNF-1. (**a**) C 1s, (**b**) N 1s, (**c**) O 1s and (**d**) Si 2p spectra.

Figure 4 and Figure S2 demonstrate the XPS pattern of SiO/CNF-1. As shown in Figure S2, the SiO/CNF-1 composite material contains four elements, referring to C, N, O and Si. Figure 4a shows the C 1s spectra. It can be seen clearly that the peak of C 1s exhibits several peaks corresponding to the C-C/C=C bond, C-N bond, C-O bond and O-C=C bond located at 284.7, 285.3, 286.5 and 288.8 eV, respectively [65]. As shown in Figure 4b, the N 1s spectra exhibit several peaks located at 398.2, 399.7, 401.0 and 402.9 eV, which are attributed to the pyridinic N, pyrrolic N, graphitic N, and oxidized N, respectively [66]. As is shown in Figure 4c, the O 1s peak can be fitted into three peaks located at 531.2, 532.2 and 533.4 eV, which should be attributed to Si=O, C=O and Si-O bonds, respectively. As for the Si 2p XPS spectra shown in Figure 4d, the Si 2p spectra can be fitted into four peaks; they are Si^0^, Si^+^, Si^2+^, and Si^3+^ located at 100.0, 101.9, 103.6 and 104.4 eV, respectively. The XPS spectra corroborate that CNF is doped with a N element after carbonization to gain effectively enhanced conductivity, which is fulfilled via the carbonization of N-contained PAN nanofibers. Additionally, the embedded SiO particles still maintained their original valence state, and did not oxidize after carbonization, manifesting excellent original stability.

**Figure 5:**
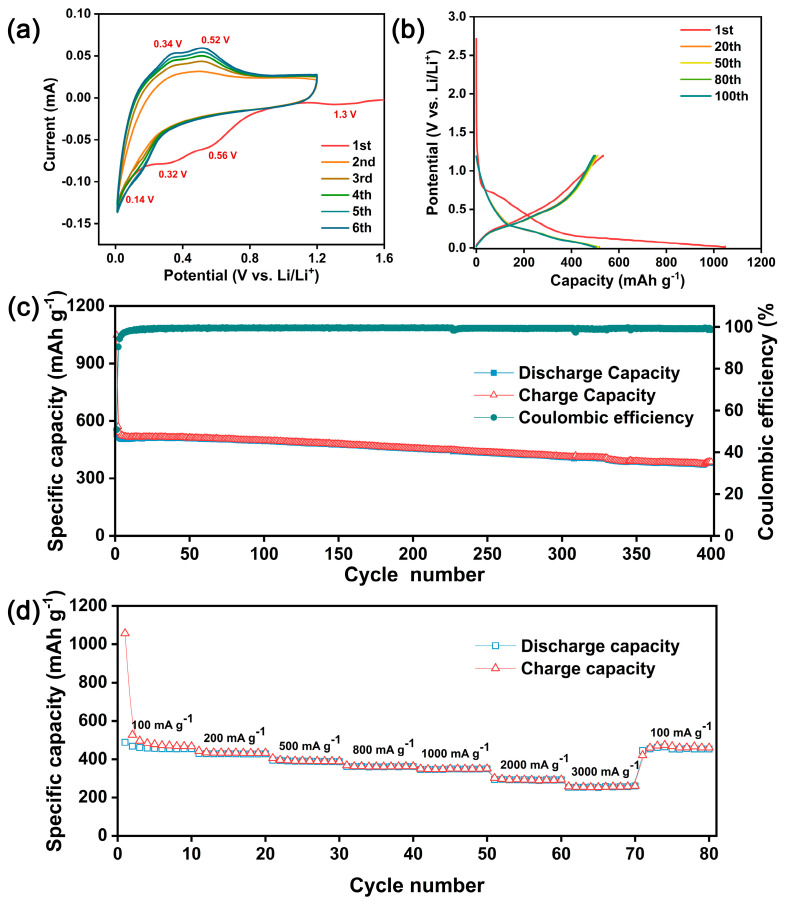
(**a**) CV curves of SiO/CNF-1; (**b**) charge–discharge potential profiles of SiO/CNF-1; (**c**) stability test of SiO/CNF-1 at a current density of 100 mA g^−1^ and (**d**) rate ability of SiO/CNF-1.

To further analyze the transmutation of electrochemical characteristics at the beginning of cyclic charge and discharge, the CV test in the potential range of 0.01 to 1.2 V at a 0.05 mV s^−1^ scan rate was conducted for SiO/CNF-1. As shown in Figure 5a, characteristic peaks of the first discharge cycle are offset from those of the subsequent discharge cycles. In the first negative sweep, the characteristic reduction peaks were located at 0.32, 0.56 and 1.3 V. The reduction peak at 0.32 V corresponds to the SiO reduction and lithium alloying process, while the reduction peaks at 0.56 and 1.3 V are related to the formation of SEI film. After the first cycle, the subsequent CV curves of SiO/CNF-1 can almost overlap. The oxidation peaks at 0.34 and 0.52 V as well as the reduction peak at 0.13 V correspond to the reversible transformation between the Li_x_Si phase and Si phase. With the increase in cycle number, the characteristic peaks’ intensity of redox becomes sharper and sharper, which is related to the increasing alloying degree of Li_x_Si; the alloying reaction of Li_x_Si formation is gradually ample as well. As for active SiO, the formation of the SEI film process contains not only the consumption of metal Li source but also involves an irreversible loss of SiO, which will produce Li_2_O and Li_x_SiO_y_.

The above characteristics can also be reflected by the relationship between voltage and capacity in the charge–discharge cycle. Figure 5b shows the charge–discharge potential profiles of SiO/CNF-1 for the 1st, 20th, 50th, 80th and 100th cycles. There is a certain capacity loss after the first charging and discharging process apparently, which represents the formation of SEI film. This loss can be expressed by Coulombic efficiency, and the initial Coulombic efficiency (ICE) of SiO/CNF-1 is 50.9%. In the voltage range of 0.25 to 0.75 V, a plateau with a slow capacity decline appears, which refers to the reduction in SiO and the formation of SEI film. In the subsequent cycles, the SEI film is no longer formed, so the charge–discharge capacity is basically the same, and the Coulombic efficiency can nearly reach 100%. Furthermore, with the increase in the cycle number, the capacity of the battery does not change significantly, which reflects the high stability of the SiO/CNF-1 electrode material during the charge–discharge cycle.

To further study the stability of the anode material we designed, the long cycle test of SiO/CNF-1 was performed at a current density of 100 mA g^−1^. As shown in Figure 5c, after 400 cycles, the discharge capacity still remains at 382 mA g^−1^, and the capacity retention rate is 73.9%. During the entire long cycle test, the Coulombic efficiency of SiO/CNF-1 can be higher than 90% just after the second cycle and keeps close to 100% after stabilizing (about 15 cycles).

Figure 5d identifies the rate performance of the SiO/CNF-1 at different current densities. The current densities of 100, 200, 500, 800, 1000, 2000 and 3000 mA g^−1^ were selected in the rate ability test, and the current density was restored to 100 mA g^−1^ at the end of the test. When the current density changes slightly, such as 100 to 1000 mA g^−1^, the capacity of the battery changes slightly, and the maximum loss is only 23% compared with the initial capacity. While the current density increases to 2000 and 3000 mA g^−1^, the capacity retention rate of the material is 62% and 56%, respectively. After the current density was adjusted back to 100 mA g^−1^, the capacity of the electrode material was restored to about 99% of its original capacity. The electrode material possesses excellent charging and discharging characteristics generally, and still performs with stable capacity recovery ability after charging and discharging at a high current density. This is supposed to be credited to the structure of SiO/CNF-1. The conductive skeleton provided by the CNF framework is the key to the stable rate performance of the electrode. Since the overall conductivity of the anode material gets better, the impedance value usually becomes lower, leading to lower energy loss during the cyclic charge and discharge and more stable capacity retention ability.

Figure S3 shows the cycling performance of different SiO/CNF samples and pure SiO anode at a current density of 100 mA g^−1^. It can be seen from the graph that for SiO/CNF samples, the capacity of charge and discharge reduced with the increase in SiO content, whilst all of them can reach or exceed the performance of the pure SiO sample. For SiO/CNF-1, SiO/CNF-2, SiO/CNF-3 and pure SiO, the initial Coulombic efficiencies are 50.9%, 48.1% 35.4% and 42.5%, respectively, and the initial discharge capacities in steady-state are 517, 392, 185 and 350 mAh g^−1^, respectively. Additionally, the SiO/CNF-1, SiO/CNF-2 SiO/CNF-3 and pure SiO anode displayed a reversible capacity of 495, 480, 270 and 269 mAh g^−1^ for over 100 repeated charge–discharge cycles, respectively. Compared with the SiO/CNF-2, SiO/CNF-3 and pure SiO, the electrochemical capacity of SiO/CNF-1 almost keeps steady and no apparent loss appears throughout the charging and discharging process. Additionally, the Coulombic efficiency is stable and high. Furthermore, the abnormal increase or drastic decay cannot be seen in the cycling curve of SiO/CNF-1. Notwithstanding, occasional performance fluctuations during charge and discharge occur among the other three-electrode materials, leading to fluctuations in Coulombic efficiency accordingly. Meanwhile, some materials have abnormal increases in capacity during the cycle. The reasons for the above phenomenon can be roughly deduced as follows. Firstly, the active material SiO agglomeration can be formed via increasing the SiO content. Compared with uniformly dispersed SiO, the size of agglomerated SiO particles becomes larger, and it is easier to shrink and expand in volume suddenly during the cycling process. Secondly, gathered active material SiO particles could not fully contact the CNF skeleton, resulting in poor conductivity and unstable electrochemical performance. Thirdly, high active material SiO concentration samples and the agglomerated SiO particles possess an enlarged particulate size; consequently, the time (or number of cycles) needed for the electrolyte solution ions to fully penetrate the SiO particles will be increased. It is also not conducive to give full play to the electrochemical performance of the electrode material, and the specific capacity of the battery will increase abnormally with the increase in the cycle number. Compared with the initial discharge capacity, after 100 cycles of test, the capacity retention rates were 95.8%, 122.6%, 145.7% and 76.8% for SiO/CNF-1, SiO/CNF-2, SiO/CNF-3 and pure SiO, respectively. Excluding the abnormal increase in capacity, SiO/CNF-1 retains a better capacity retention rate compared with pure SiO. The discharge capacity of SiO/CNF-1 after stabilization is 1.47 times that of pure SiO, and the capacity of SiO/CNF-1 after 100 cycles is 1.84 times that of pure SiO. The excellent properties can be emphasized as follows. On the one hand, CNF provides a conductive framework for SiO, and the conductivity of SiO/CNF is greatly improved to ensure the full intercalation and de-intercalation of Li^+^ ions during the charging and discharging process. On the other hand, SiO particles are buddled by CNF, and the structure can effectively tolerate the volume variation in SiO during the cyclic process, which ensures that the SiO will not be inactivated due to volume expansion and lead to capacity attenuation.

**Figure 6:**
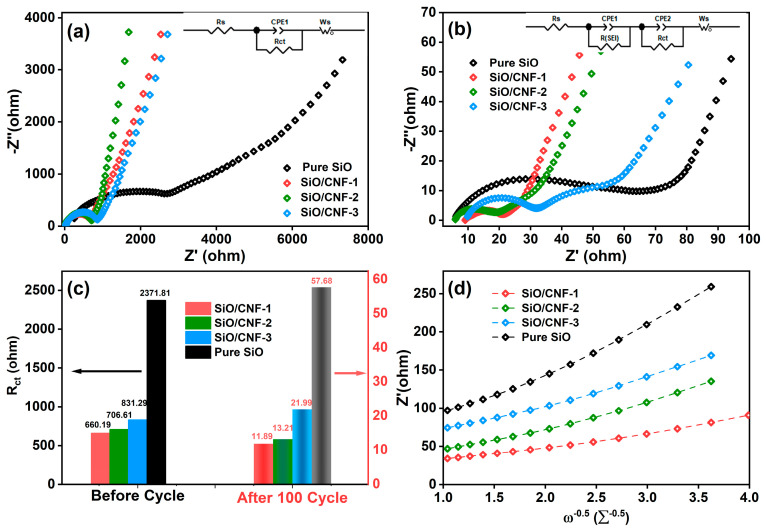
Kinetics analysis of SiO/CNF and pure SiO electrodes. EIS of SiO/CNF (**a**) before cycle and (**b**) after 100th cycle. (**c**) Simulation results of charge-resistant R_ct_ shown in (**a**,**b**), (**d**) SiO/CNF and pure SiO Z’ vs. ω^−0.5^ after cycling.

As shown in Figure 6, the kinetic analyses of the battery before and after cyclic charge and discharge were conducted by electrochemical impedance spectroscopy. Figure 6a,b exhibit the EIS pattern of the SiO/CNF anode before and after the 100th cycle. Different intervals correspond to different resistance values. The first intersection of the EIS curve and Z’ axis represents the resistance value of electrolytes (R_S_), the semicircle represents the value transfer resistant (R_ct_) between the electrode and electrolyte, and the sloping line represents the diffusion resistance of Li^+^ ions [67,68]. It can be observed from Figure 6a that there is only one semicircle in the high-frequency regions and a line in the low-frequency region for all SiO/CNF-1, SiO/CNF-2, SiO/CNF-3 and pure SiO before activation. The high-frequency range semicircle is related to the charge-transfer impedance (R_ct_) at the electrode interface. At a lower frequency, the linear region is contributed to the Li^+^ ion diffusion into the active materials. However, it can be seen from Figure 6b that the Nyquist plots include two depressed semicircles in the middle and high frequencies after the 100th cycle, which should be ascribed to the formation of SEI after activation, and a linear tail in the low frequency. Before activation, the charge-transfer impedance of SiO/CNF electrode is much lower than that of SiO electrode. It is noteworthy in Figure 6c that the Rct values of samples reduce from 660.19, 706.61 and 831.29 Ω to 11.889, 13.212 and 21.994 Ω for SiO/CNF-1, SiO/CNF-2 and SiO/CNF-3, respectively, while the value of pure SiO decreases from 2371.8 to 57.677 Ω. The corresponding equivalent circuits are represented by the inset in Figure 6a,b, respectively. The fitted values of R_s_ and R_ct_ of all samples are demonstrated in Table 3. After 100 cycles, the R_ct_ values of all samples decrease with the different magnitudes. The R_ct_ of SiO/CNF-1 after 100 cycles was 11.89 Ω, the lowest among all samples, indicating the fastest charge-transfer kinetics, which is contingent on the judicious fibrous structure of SiO/CNF-1.

Figure 6d shows the relation between *Z′_real_* and *ω*^−0.5^. The relation was fitted according to the following equation [69,70,71]

(1) $$Z_{real}^{’}=R_{s}+R_{SEI}+R_{ct}+\sigma_{\omega }\omega^{-0.5}$$

where *ω* is the angular frequency and *σ_ω_* is the Warburg coefficient. For all SiO/CNF and SiO anodes, *σ_ω_* can be calculated as 18.35, 32.42, 35 and 62.08 Ω s^−0.5^, respectively. The following equation is used to express the diffusion coefficient of Li^+^ ions (D) [52,69,72]

(2) $$D=0.5{\left(\frac{RT}{AF^{2}n^{2}C\sigma_{\omega }}\right)}^{2}$$

where *R*, *T*, *A*, *F*, *n*, and *C* are the gas constant, temperature, anode surface, Faraday’s constant, the number of electrons and the Li+ ion concentration, respectively. According to Equations (1) and (2), *σ_ω_* and *D* are calculated as shown in Table 1. It can be disclosed clearly that the value D of the SiO/CNF anode is higher than that of pure SiO. Among all the SiO/CNF samples, SiO/CNF-1 outperforms with the largest value of D (8.30 × 10^−15^ cm^2^s^−1^). The largest D value can render SiO/CNF with outstanding structural privilege within all samples towards Li^+^ diffusion inside the anode material. The SiO/CNF not only militates against the volume changes in active material SiO, but also constructs Li^+^ ion transport channels.

Table 4 shows the electrochemical performances of SiO-based anode materials. Compared with the previously reported SiO-based anode materials, SiO/CNF has a better capacity retention rate, which is benefited from the unique and conductive carbonaceous skeleton generated via electrospinning. However, the capacity and ICE still need to be improved.

## 4. Conclusions

In summary, an electrospun high-performance SiO/CNF anode material for LIBs has been designed and fabricated via a cost-effective and environmentally benign method. This excellent structure not only provides a conductive network for active material SiO nanoparticles but also surmounts the extensive reunion sufficiently. The excellent SiO/CNF structure exhibits a prominent electrochemical performance compared with pure commercial SiO. SiO/CNF remains to have 73.9% of initial capacity after 400 cycles at a current density of 100 mA g^−1^. Furthermore, the diffusion coefficient of SiO/CNF was found to be ~8 × 10^−15^ cm^2^ s^−1^, which is more than 10 times higher than pure SiO material. All these above can corroborate that the electrochemical performance of raw SiO particles was significantly modified via the facile and green electrospinning method. Boosted by a unique and conductive carbonaceous skeleton generated via electrospinning, this high-performance SiO/CNF anode material demonstrates great potential of being central to the solution of the commercial applications of LIBs.

## Figures and Tables

**Table 1:** Comparison of various Si-based anode materials for the related factors in lithium-ion batteries.

Materials	Si	SiO	SiO_2_
Capacity (mAh g^−1^)	4200 [34]	2600 [35]	1965 [36]
Coulombic Efficiency (%)	100 [37]	69 [38]	52 [39]
Charge–Discharge Rate	High	Medium	Low
Volume Expansion (%)	300	150	100
Safety [40]	Low	Medium	High
Cost [41]	High	Medium	Low

**Table 2:** Electrochemical performance of SiO composites in half-cell testing.

Anode Material	Current Density/Cycles/Cycle Stability	Initial Coulombic Efficiency	Capacity Retention	References
Boron-doped SiO	0.5 C/100/947 mAh g^−1^	65.1%	90%	[45]
Li_x_Si/Li_2_O	0.5C/400/961 mAh g^−1^	77.7%	99.87%	[44]
SiO/Fe_2_O_3_	160 mA/g/50/133 mAh g^−1^	68%	71%	[47]

**Table 3:** Fitted values of Rs, R_ct_ RSEI, σ_ω_ and D of SiO/CNF.

Samples	Before Cycling		After Cycling		σ_ω_ (Ω s^−0.5^)	D (cm^2^s^−1^)
	R_s_ (Ω)	R_ct_ (Ω)	R_s_ (Ω)	R_ct_ (Ω)		
SiO/CNF-1	20.01	660.19	9.10	11.89	18.35	8.30 × 10^−15^
SiO/CNF-2	5.59	706.61	5.95	13.21	32.42	2.66 × 10^−15^
SiO/CNF-3	29.71	831.29	9.86	21.99	35.00	2.28 × 10^−15^
SiO	252.20	2371.80	6.15	57.68	62.08	0.73 × 10^−15^

**Table 4:** Electrochemical performances of SiO-based anode materials.

Materials	Charge Capacity (mA h g^−1^)	ICE(%)	Current Density	Cycling Performance (mA h g^−1^)	Capacity Retention Rate (%)	Ref.
Bm-SiO/Ni/rGO	1636.4	62.4	0.1 A g^−1^	720.3 (100 cycles)	70.5	[72]
SiO/Fe_2_O_3_	1893	68	4.8 A g^−1^	~1335 (50 cycles)	71	[46]
SiO-C	1491	64.81	0.1 C	1000 (100 cycles)	67.2	[73]
7LTO3SiO	621.3	94.82	1 A g^−1^	228.7 (100 cycles)	36.8	[74]
VGCF/Super P	600.6	87.9	0.2 C	403 (40 cycles)	67.1	[75]
SiOx/C@Graphite	591.2	40.16	1 A g^−1^	374.5 (100 cycles)	63.3	[76]
SiO/CNF	516.5	50.9	0.1 A g^−1^	381.7 (400 cycles)	73.9	This work

## Data Availability

The authors confirm that the data supporting the findings of this study are available within the article.

## Supplementary Materials

The following supporting information can be downloaded at https://www.mdpi.com/article/10.3390/nano14141223/s1. Figure S1: SEM images of SiO particles (a–c) before and (d,e) after ball-milling, and the diameter distribution histograms (g) before and (h) after ball-milling; Figure S2: XPS survey spectra of SiO/CNF-1; Figure S3: Charge–discharge properties of various samples at a current density of 100 mA g^−1^. (a) SiO/CNF-1, (b) SiO/CNF-2, (c) SiO/CNF-3, and (d) SiO.
