# Supplementary material for: A Hybrid Structure to Improve Electrochemical Performance of SiO Anode Materials in Lithium-Ion Battery
Source: Nanomaterials (Basel). 2024 Jul 19;14(14):1223. doi: 10.3390/nano14141223 (PMC11279576; doi:10.3390/nano14141223)
Supplement: Supplementary file 1 [file nanomaterials-14-01223-s001.zip › nanomaterials-3064203-supplementary.pdf]

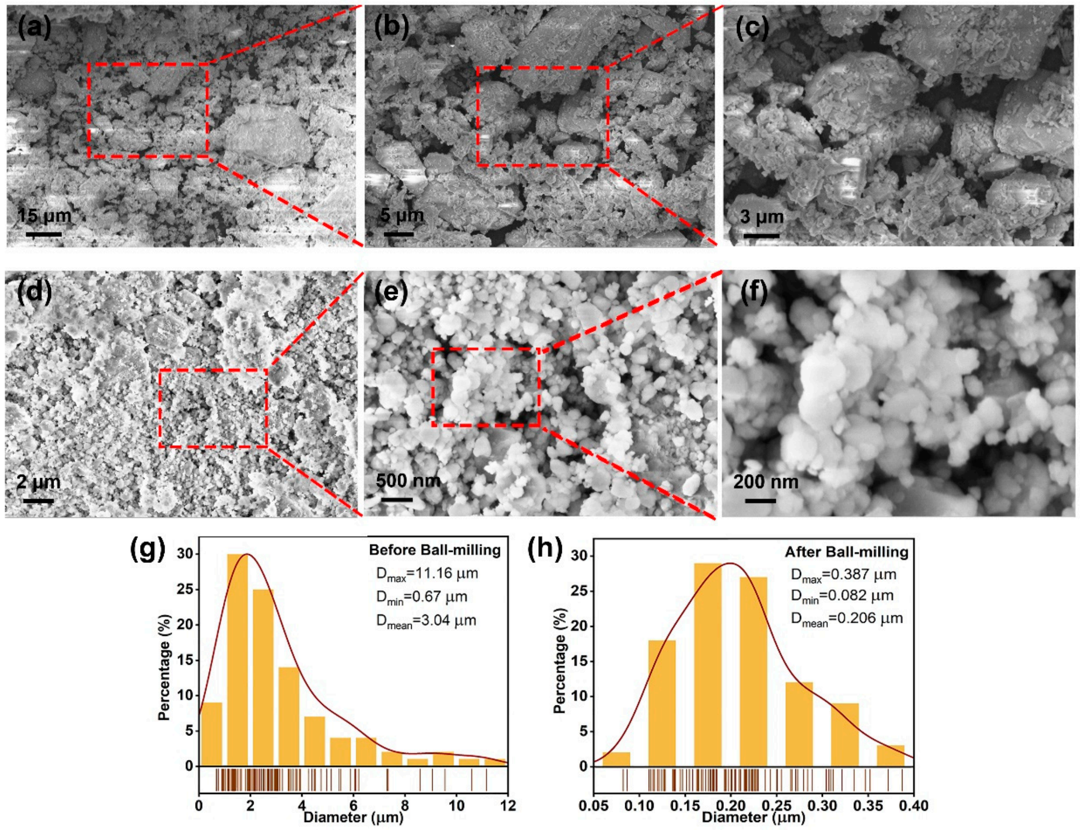

Figure S1. SEM images of SiO particles (a-c) before and (d-e) after ball-milling, and the diameter distribution histograms (g) before and (h) after ball-milling.

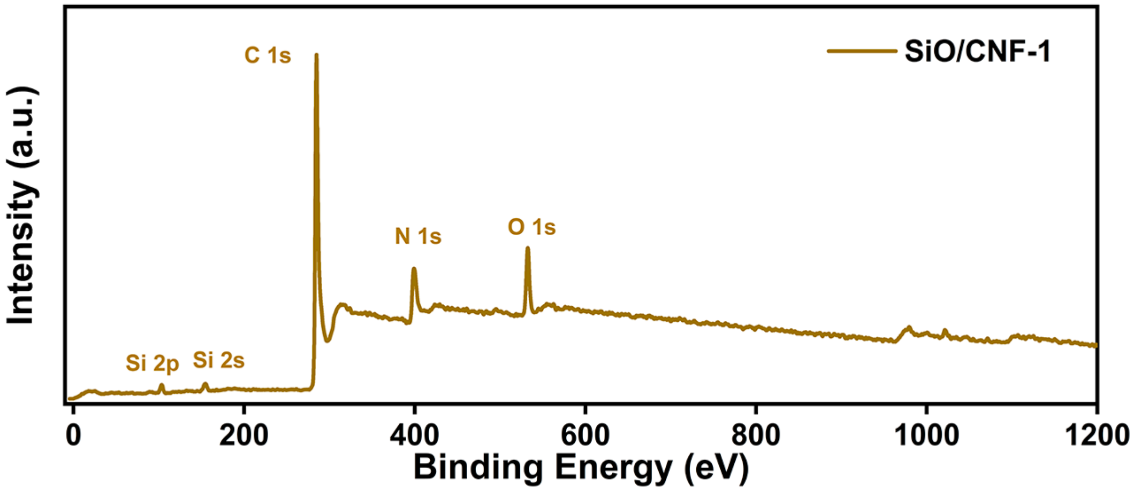

Figure S2. XPS survey spectra of SiO/CNF-1.

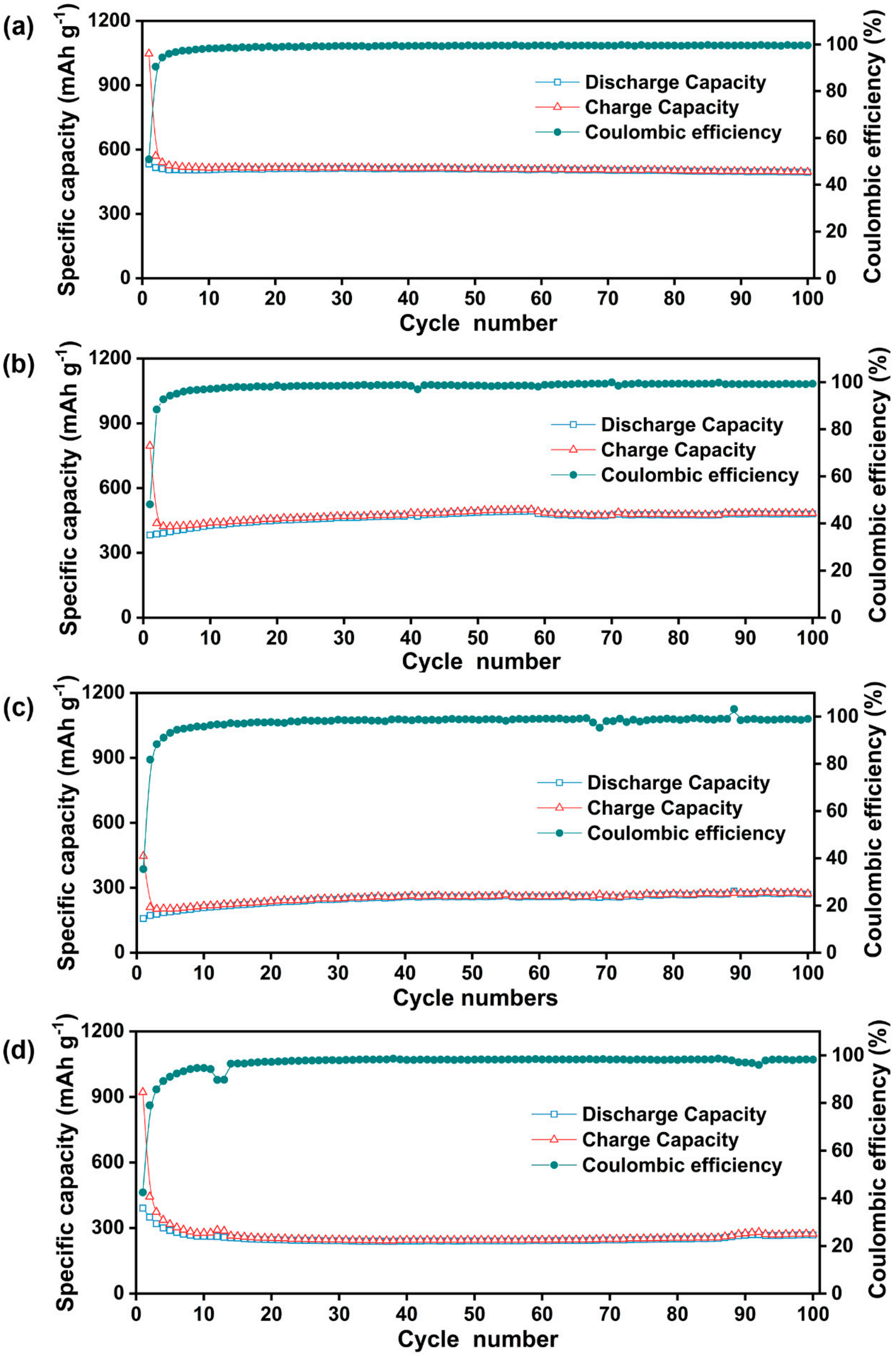

Figure S3. Charge-discharge properties of various samples at a current density of 100 mA g^{-1} (a) SiO/CNF-1, (b) SiO/CNF-2, (c) SiO/CNF-3, (d) SiO.
